# Hypolipidemic effect of *Alisma orientale* (Sam.) Juzep on gut microecology and liver transcriptome in diabetic rats

**DOI:** 10.1371/journal.pone.0240616

**Published:** 2020-10-09

**Authors:** Xiaomei Xu, Lisha Li, Yamin Zhang, Xuehua Lu, Wei Lin, Shuangshuang Wu, Xia Qin, Rongqing Xu, Wenjin Lin

**Affiliations:** 1 Fujian Key Laboratory of Medical Measurement, Fujian Academy of Medical Sciences, Fuzhou, China; 2 Department of Endocrinology, Shengli Clinical Medical College of Fujian Medical University, Fujian Provincial Hospital, Fuzhou, China; East Tennessee State University, UNITED STATES

## Abstract

*Alisma orientale* (Sam.) Juzep (*A*. *orientale*) is a traditional herb that is often used to treat disease including edema and hyperlipidemia. However, the molecular mechanism by which *Alisma orientale* (Sam.) Juzep exerts its hypolipidemic effects remains unclear. In this study, a diabetic rat model was established by feeding a high-fat and high-sugar diet combined with a low-dose streptozotocin injection (HFS). Then the rats were treated with an *A*. *orientale* water extract (AOW), an *A*. *orientale* ethanolic extract (AOE) or metform (MET). The gut microflora and liver transcriptome were analyzed by high-throughput next-generation sequencing. Ultra-performance liquid chromatography-triple quadrupole-mass spectrometry was employed to analyze the major compounds in the AOE. The results showed that the serum total cholesterol (TC) and low density lipoprotein cholesterol (LDL-C) levels in rats of the AOE group (2.10 g/kg/day, 14 days) were significantly lower than those in the HFS group (*p*<0.01). Moreover, AOE treatment altered the gut microecology, particularly modulating the relative abundance of gut microflora involved in lipid metabolism compared with the HFS group. Furthermore, compared with the HFS group, the mRNA expression levels of Fam13a, Mapk7, Mpp7, Chac1, Insig1, Mcpt10, Noct, Greb1l, Fabp12 and Hba-a3 were upregulated after the administration of AOE. In contrast, the mRNA expression levels of Lox, Mybl1, Arrdc3, Cyp4a2, Krt20, Vxn, Ggt1, Nr1d1 and S100a9 were downregulated. Moreover, AOE treatment for two weeks markedly promoted the relative abundance of *Lachnospiraceae* (*p* = 0.0013). The triterpenoids contents in AOE were alisol A, alisol A 24-acetate, alisol B, alisol B 23-acetate, alisol C 23-acetate, alisol F, alisol F 24-acetate, and alisol G. Our findings above illustrated that the hypolipidemic effect of the triterpenoids of *A*. *orientale* is mediated mainly through alteration of the gut microecology and the regulation of genes involved in cholesterol metabolism, especially Insig1.

## Introduction

Diabetes mellitus (DM) is a metabolic disease associated with the inability to produce insulin and the inability to properly utilize glucose (T1D) or insulin resistance when interacting with its receptor (T2D) [1]. Approximately 10% of the world’s population already has T2D or may develop it, and approximately 40% of adults are overweight or obese [2]. Obesity is a major risk factor for metabolic syndrome and T2D, which share a potential mechanism of insulin resistance (IR) [3, 4]. So far, many antihyperglycemic chemical or biological drugs have been developed, such as insulin secretagogues (Thiazolidinediones, Sulfonylureas, Biguanides), insulin sensitizer agents, α-glucosidase inhibitors, GLP-1 analogs, DPP-4 inhibitors, cannabinoid-1 receptor inhibitors, G protein-coupled receptor 40 agonists and GPR119 agonists [5]. However, some inevitable adverse effects, such as hypoglycemia, liver dysfunction and gastrointestinal reactions, have attracted considerable attention [5].

DM is known as Xiao-Ke disease in ancient China in terms of polydipsia, polyphagia, polyuria, and body weight loss. Traditional Chinese medicines were used to treat the disease for around 1, 500 years [6]. In recent years, increasing amounts of researches have shown that some Chinese herbs or their extracts can effectively treat T2D and/or obesity [7, 8]. Iridoid glycoside from *Cornus officinalis* effectively mitigated the general symptoms of DM rats including weight loss, polydipsia, polyphagia, polyuria, elevated blood glucose level and low serum insulin level [9]. Purified anthraquinone-glycoside from *Rheum palmatum* L. could reduce oxidative stress in rats with DM by improving their blood lipid metabolism and enhancing their antioxidant capacity, thereby inhibiting beta-cell apoptosis and improving beta-cell function [10, 11]. Quercetin extracted from the flowers of *Edgeworthia gardneri* exerted excellent properties in islet protection and amelioration [12]. Lychee seed extract remarkably prevented neuronal injury and improved cognitive functions in T2DM rats [13]. The two fractions of *Morus alba* fruit polysaccharides (MFP50 and MFP90) had markedly antihyperglycemic and antihyperlipidemic effects and could relieve diabetes symptoms in the T2D rat model [14]. Hawthorn extracts decreased the levels of triglyceride, total cholesterol and blood glucose, whereas the level of plasma insulin released from the pancreas was increased [15]. In addition, Zhang et al. showed that the ethyl acetate extract of *Forsythia suspense* (Thunb.) Vahl fruit could treat DM via modulation of oxidative stress, hepatic glucose metabolism and pancreatic insulin secretion [16].

*Alisma orientale* (Sam.) Juzep (*A*. *orientale*) is a well-known medical plant of the *Alisma* genus and *Alismataceae* family in China, and is also distributed in the former Soviet Union, Mongolia, Korea and Japan. *A*. *orientale* is sweet in taste and cold in properties. It is mainly used to treat wind, cold and dampness impediments and lactation difficulties, to eliminate water, to nourish the five Zang-organs, to increase energy and to strengthen the body. It is effective in promoting urination, eliminating dampness and resolving heat. Clinically, it is used to treat dysuria, edema, beriberi, diarrhea, stranguria and leucorrhea.

The Liuwei Dihuang decoction is a well-known classic traditional Chinese medicine formula, which consists of six herbs including *A*. *orientale*. It can intervene in the insulin resistance of T2DM, in part through regulation of the canonical PI3K/Akt signaling pathway [17]. Both *A*. *orientale* and gliclazide monotherapy are effective in lowering fasting blood glucose. As a single-target drug, gliclazide has a stronger effect in lowering fasting glucose. However, *A*. *orientale* can also control weight and improve glucose intolerance [18].

Kim et al. [19] have reported metabolomic changes in the evaluation of the treatment effects of *A*. *orientale* and *Artemisia capillaris* on fatty livers in diabetic mice. The AMP/ATP ratio of cellular energy homeostasis in *A*. *orientale*-treated mice was increased relative to than that of the *Artemisia capillaris*-treated mice. *A*. *orientale* is a more effective natural product in the treatment of liver dysfunction than *Artemisia capillaris*. Unfortunately, they did not report the hypoglycemic effect of *A*. *orientale* and *Artemisia capillaris* in diabetic mice.

In this study, in order to explore the hypoglycemic and hypolipidemic effects of *A*. *orientale* and its potential molecular mechanism, a DM rat model was established. Subsequently, the water extract (AOW) and ethanolic extract (AOE) of *A*. *orientale* were separately administered for hypoglycemic and hypolipidemic treatment. The liver is an important organ that plays a central role in the regulation of systemic glucose and lipid metabolism [19]. What’s more, the microbiota also affects many metabolic functions, including the regulation of glucose and lipid homeostasis. There is increasing evidence that any modification of the microbiota composition may lead to a variety of diseases, including metabolic diseases, such as diabetes, obesity, and cardiovascular diseases [20–22]. In view of the above factors, the serum biochemical indicators, liver histopathology observation, liver transcriptome analysis and gut microflora were evaluated. The gut microflora and liver transcriptome were analyzed by high-throughput next-generation sequencing. This is the first report of the regulatory efficacy of *A*. *orientale* on gut microecology and the liver transcriptome profile in vivo.

## Materials and methods

### Ethics statement

The study was approved by the ethics review board of Fujian Academy of Medical Sciences (Fuzhou, Fujian Province, China). All experimental procedures were performed in accordance with the China legislation regarding Laboratory animal—Guideline for ethical review of animal welfare (GB/T 35892–2018) published by the General Administration of Quality Supervision, Inspection and Quarantine of the People’s Republic of China (02-06-2018 issued, 09-01-2018 implemented). All surgery was performed under anesthesia, and all efforts were made to alleviate suffering.

### Preparation of the *Alisma orientalis* extract

The dried samples of *A*. *orientale* were provided by Zhuqing Lv at the Station for Popularizing Agricultural Technique of Nanping City, Fujian Province, China. 252 g of *A*. *orientale* was boiled in 5040 ml (1:20) of 80% ethanol or water twice, 1.5 hours each time, using the method of reflux extraction. The extracts were then combined, filtered and concentrated to a density of 0.21 g crude herb/ml by a rotary evaporator, and stored at 4°C until further use.

### Establishment of diabetic rat model and experimental design

Forty-eight specific pathogen-free male Sprague Dawley (SD) rats (eight weeks old, 180–220 g) used in this experiment were purchased from the Shanghai Laboratory Animal Company (SLAC, Shanghai, China). All rats were housed in sanitized polypropylene cages in a hygienic room with a natural day-night light cycle with a regulated temperature (23–26°C) and 55–65% relative humidity. Standard water and chow diet were provided ad libitum.

After a week of adaptive feeding, the rats were randomly divided into a normal diet group (n = 16) and high fat diet group (n = 32). Rats in the normal diet group were fed with standard rodent chow, while those in the high fat diet group were fed with a high-fat diet containing 60% energy from fat for four weeks (Trophic Animal Feed High-Tech Co., Ltd., Nantong, China). Their food intake was detected daily and the body weight was measured once a week.

On day 29, we fast all rats for 6 hours (from 7:00 a.m. until 1:00 p.m.) prior to STZ treatment. Water was provided as normal. The experimental rats were injected with STZ intraperitoneally at 40 mg/kg in 0.1 M citrate buffer (pH 4.5). The control rats were given an equal dose of citrate buffer [23]. Three days after STZ administration, we measured their blood glucose level in a tail-vein blood sample using a One Touch Basic blood glucose monitoring system. Rats with a fasting blood glucose range of 11–14 mmol/L were considered T2DM and subsequently used for the study [24].

The rats fed a high-fat diet with STZ intraperitoneally injected were then randomly divided into four groups: the first group was orally administered saline as a model control (HFS group, n = 8), while the second group was orally administered metformin as a positive control at 250mg/ (kg·d) (MET group, n = 8). The third group was orally administered the water extract of *A*. *orientale* at 2.1g/ (kg·d) (AOW group, n = 8), while the fourth group was orally administered the ethanol extract of *A*. *orientale* at 2.1g/ (kg·d) (AOE group, n = 8). The dose of 2.1g/(kg·d) was decided basing on twice the usual clinical dose. For the low fat diet group, the eight rats intraperitoneally injected with STZ only were classified as the STZ group, while the remaining eight rats intraperitoneally injected with citrate buffer were used as a control group (CON group).

### Biochemical measurements of the serum

After two weeks of treatment, all rats were kept on a fast for 12 hours. The rats were then sacrificed under anesthesia with pentobarbital sodium at a dose of 40 mg/kg, and blood was obtained from the abdominal aorta. The Serum was separated by centrifugation at 3000rpm for 15 min at 4°C. The serum aspartate transaminase (AST), alanine aminotransferase (ALT), triglyceride (TG), total cholesterol (TC), low-density-lipoprotein cholesterol (LDL-C), and high-density-lipoprotein cholesterol (HDL-C) levels were measured by an automatic biochemical analyzer (BS-400 MINDRAY Chemistry Analyzer, China).

### Liver and cecal content samples collection

Right after the blood collection, the livers were removed and weighed, then washed with normal saline. The partial liver tissues of the right lobe were excised and fixed in 10% formaldehyde solution for paraffin embedded sections. The remaining parts of the liver tissues were immediately cut into small pieces and frozen in liquid nitrogen for approximately 15 min then stored at −80 °C until liver transcriptome and Q-PCR determination. Simultaneously, the tissues of the cecum were cut and the cecal content samples were collected into a cryotube and frozen in liquid nitrogen for approximately 15 min then stored at −80 °C for throughput sequencing analysis of the gut microbiota.

### Liver histopathological observation

For histopathological observation, the collected liver samples fixed in formaldehyde solution were treated with a series of different concentrations of ethanol solutions. The liver tissues samples were embedded in paraffin and cut into thick sections by a pathological slicer, then stained with hematoxylin-eosin (H&E) and oil red O, and liver histopathological observation were performed under an optical microscope.

### Gut microbiota total DNA extraction and high throughput sequencing

Three samples from the gut microbiota were collected randomly from each of the groups. The total DNA was extracted from the cecal contents of the rats using the QIAamp Fast DNA Stool mini kit (Cat ID: 51604, Qiagen, Germany) according to the manufacturer’s instructions. The V3-V4 hypervariable regions of 16S rRNA from the gut microbiota were amplified using standard primers (forward primer 5′-ACTCCTACGGGAGGCAGCA-3′ and reverse primer 5′- GGACTACHVGGGTWTCTAAT-3′). Sequencing libraries were prepared using Illumina’s TruSeq Nano DNA LT Library Prep Kit. The quality of the libraries was tested on the Agilent Bioanalyzer using the Agilent High Sensitivity DNA Kit (Agilent, USA). The libraries were quantified on a Promega QuantiFluor fluorescence quantification system using the Quant-iT PicoGreen dsDNA Assay Kit (Thermo Fisher Scientific, USA). The sequencing was performed on the Illumina MiSeq instrument with a 2 × 300 paired-end configuration using the MiSeq Reagent Kit V3 (Illumina, USA).

### Microbiota diversity analysis

To explore the diversity of the microbiota species composition, the paired-end sequences were paired according to overlapping bases using the FLASH software (v1.2.7, http://ccb.jhu.edu/software/FLASH/) [25], and the connected paired sequences were assigned to the corresponding samples according to the barcode sequences, thereby obtaining an effective sequence of each sample. Operational taxonomic units (OTUs) were generated by clustering at a similarity of 97% using the QIIME software and the UCLUST sequence alignment tool [26] against the Greengenes database [27], RDP (Ribosomal Database Project) database [28] and the Silva database [29]. The alpha diversity of each sample was calculated using the Chao1 estimator [30], the ACE estimator [31], the Shannon diversity index [32], and the Simpson index [33]. The composition and abundance distribution of each sample at the five classification levels of phylum, class, order, family and genus were obtained using QIIME software. Beta diversity analysis was performed by the principal component analysis (PCA), multidimensional scaling (MDS) and clustering analysis methods. The Spearman rank correlation coefficient between the dominant genera was calculated using Mothur software. The associated network was constructed and visualized in Cytoscape (http://www.Cytoscape.org/) [34]. The PICRUSt (phylogenetic investigation of communities by reconstruction of unobserved states) was used to predict the bacterial metabolic function [35].

### Total RNA extraction and RNA-seq analysis

Five liver samples were collected randomly from each of the CON, HFS, MET and AOE groups. Total RNA was extracted using TRIzol reagent (Invitrogen Life Technologies, USA) according to the manufacturer’s protocol. The same RNA samples were used for qRT-PCR analysis. The concentration, quality and integrity were determined using a BioDrop spectrophotometer (BioDrop Technologies, UK) and an Agilent bioanalyzer 2100 (Agilent, USA). Sequencing libraries were constructed using the TruSeq RNA sample preparation kit (Illumina, USA). Briefly, mRNA was purified from total RNA using poly-T oligo-attached magnetic beads. Fragmentation was carried out using divalent cations under an elevated temperature in an Illumina proprietary fragmentation buffer. First strand cDNA was synthesized using random oligonucleotides and SuperScript II. Second strand cDNA synthesis was subsequently performed using DNA polymerase I and RNase H. The remaining overhangs were converted into blunt ends via exonuclease/polymerase activity and the enzymes were removed. After adenylation of the 3′ ends of the DNA fragments, Illumina PE adapter oligonucleotides were ligated to prepare for hybridization. To select cDNA fragments of the preferred 200 bp length, the library fragments were purified using the AMPure XP system (Beckman Coulter, Beverly, CA, USA). DNA fragments with ligated adaptor molecules on both ends were selectively enriched using an Illumina PCR primer cocktail in a 15-cycle PCR. Products were purified (AMPure XP system) and quantified using an Agilent high sensitivity DNA assay on a Bioanalyzer 2100 system (Agilent). The sequencing library was then sequenced on a Hiseq platform (Illumina) by Shanghai Personal Biotechnology Co. Ltd.

### Bioinformatic analysis

After the raw data collation, filtering and quality assessment, the filtered reads were mapped to the reference genome using HISAT2 software (http://ccb.jhu.edu/software/hisat2/index.shtml). The gene expression was normalized using FPKM, which is the number of Fragments Per Kilo bases per Million fragments. Difference analysis of gene expression was performed by DESeq, and the differentially expressed genes (DEGs) were screened with |log2 Fold Change| > 1, p <0.05. GO-enrichment analysis was performed using topGO, and the *P*-value was calculated by the hypergeometric distribution test. Pathway-enrichment analysis was performed using the Kyoto Encyclopedia of Genes and Genomes (KEGG) database annotation. The results of the differentially expressed genes, and the significance value of the differential gene enrichment was p<0.05. Protein and protein interacting (PPI) analysis was performed using STRING (Search3 Tool for the Retrieval of Interacting Genes/Proteins). The correlations heatmap was drawn using the OmicShare (http://www.omicshare.com/tools/Home/Soft/senior) online tool.

### qRT-PCR validation

To validate the Illumina sequencing data, Insig1 was selected for qRT-PCR analysis, using a SYBR Green PCR Kit (TaKaRa, Dalian, China) with 100 ng of cDNA as a template. After mixing, the PCR was performed using a LightCycler 480 instrument. The Gapdh gene was used as an internal control, and the relative gene expression level was analyzed using the 2^-△△CT^ method. The primers were synthesized by Biosune Biotech (Shanghai) Co., Ltd. Primer sequences will be provided upon request.

### Determination of the contents of triterpenoids in *A*. *orientale*

AOW and AOE samples were added to an equal amount of acetonitrile, ultrasonically mixed, then filtered through a 0.45μm filter membrane for content determination. Alisol A, alisol A 24-acetate, alisol B, alisol B 23-acetate, alisol C 23-acetate, alisol F, alisol F 24-acetate, and alisol G (MANSITE Biotechnology Co. Ltd., Chendu, China.) were selected as reference substances. Quantitative analysis of eight triterpenoids was performed on the Waters TQ TQS system of ultra high-performance liquid chromatography configured with a triple quadruple mass spectrometer (UPLC-QQQ-MS, Waters, UK), according to the methods used in previous research [36].

### Statistical analysis

All of the experimental data are expressed as the mean ± standard deviations. Statistically significant differences between the groups were analyzed by one-way analysis of variance (ANOVA) followed by post hoc multiple comparison Fisher’s LSD t-tests using SPSS 18.0 software (IBM, USA). Statistically significant differences were defined as *p*< 0.05.

## Results

### Effects of *A*. *orientale* on serum lipid and FBG levels in diabetic rats

**Fig 1:**
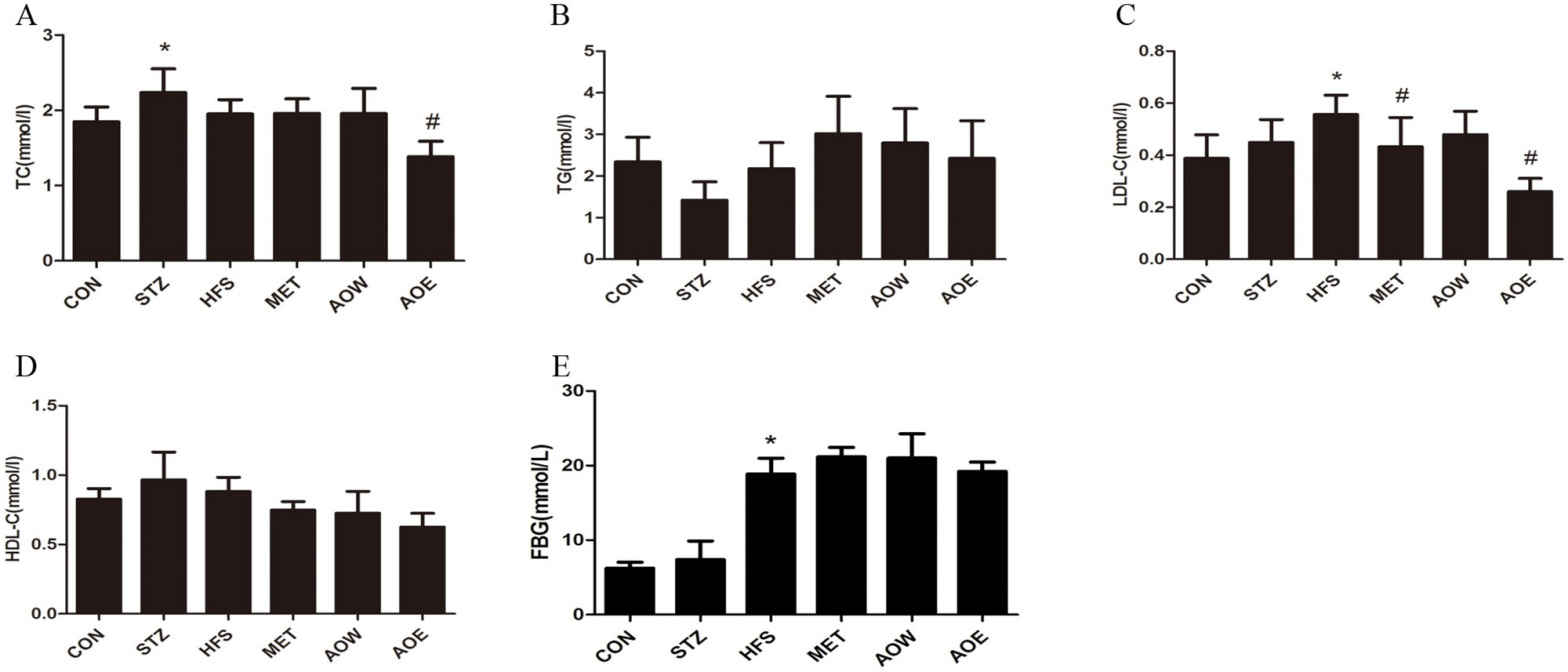
Effects of *A*. *orientale* on the levels of serum biochemical parameters and blood glucose in diabetic rats. (A) The serum level of TC. (B) The serum level of TG. (C) The serum level of LDL-C. (D) The serum level of HDL-C. (E) The level of blood glucose. Data represent the mean±SEM (n = 8). * *p* < 0.05 vs. CON group, ^*#*^ *p* < 0.05 vs. HFS group. TC, total cholesterol; TG, total triglyceride; LDL-C, low density lipoprotein cholesterol; HDL-C, high-density-lipoprotein cholesterol.

After treatment with metformin or *A*. *orientale* for two weeks, the serum level of LDL-C in the AOE group was markedly reduced compared with the HFS group. Compared to the CON group, the level of TC was increased in response to the HFS, which was then attenuated by AOE feeding. However, the levels of HDL-C, TG and FBG were not ultimately altered by the administration of metformin or *A*. *orientale* (Fig 1).

### Effect of *A*. *orientale* on liver weigh, liver function and histopathology in diabetic rats

**Fig 2:**
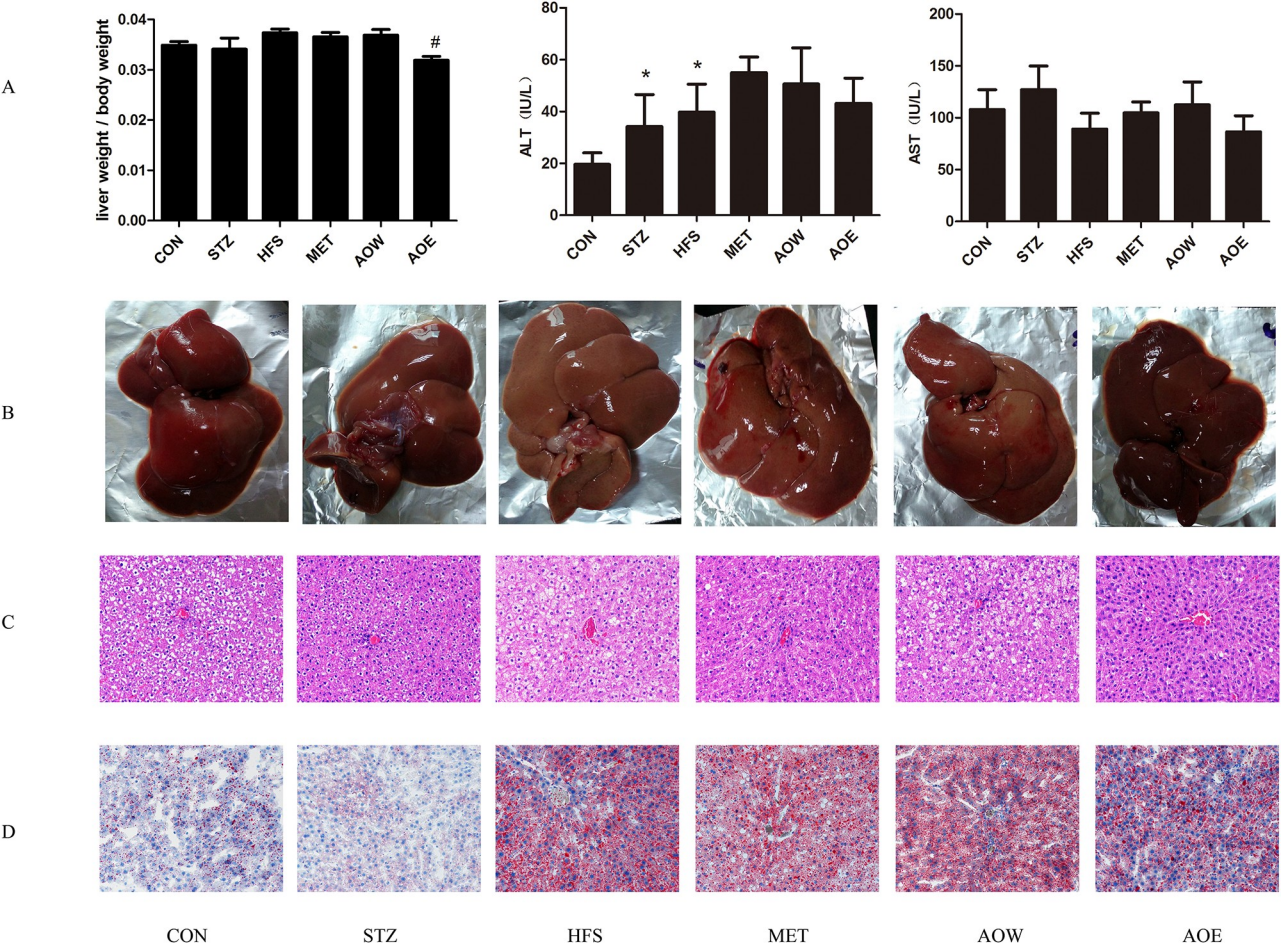
Effect of *A*. *orientale* on the ratio of liver weight to body weight, liver function and histopathology in diabetic rats. (A) The ratio of liver weight to body weight and the serum levels of ALT and AST. (B) The gross appearance of the rat livers. (C) Histopathological observations of the rat livers. H&E stained sections were photographed at 200×magnification, n = 3. (D) The Oil Red O staining of the rat livers. Data represent the mean±SEM (n = 8). * *p* < 0.05 vs. CON group, ^#^ *p* < 0.05 vs. HFS group. AST, aspartate transaminase; ALT, alanine aminotransferase.

There were no significant differences in the weight of livers between the HFS group and the CON group. The rats in the HFS group had dramatically higher levels of ALT compared with those in the CON group, which indicated that rats in the HFS group had severe liver damage. However, after treatment with metformin or *A*. *orientale*, no significant improvements were found. Moreover, no significant changes in the level of AST were observed among the different groups (Fig 2A).

The liver tissues in the CON group were normal in appearance, soft in texture, and dark red in color (Fig 2B). H&E staining showed that the liver of this group was observed to be complete, and the structure of the liver lobules was normal (Fig 2C). The results of Oil Red O staining revealed that the liver cells were not affected by steatosis (Fig 2D). The gross appearance of the liver in the HFS group was different from that of the CON group. The liver volume of the HFS group was significantly increased, the texture was brittle, and the color was greasy yellow. Correspondingly, HE staining showed that the hepatic cord structure was disordered and irregularly arranged. Most hepatocytes were swollen with severe steatosis. Moreover, an uneven size and quantity of lipid droplets were visible in the cytoplasm after of Oil Red O staining. However, after treatment with metformin or *A*. *orientale*, all of these symptoms were obviously ameliorated. In conclusion, these results indicated that *A*. *orientale* might ameliorate HFS-induced hepatic steatosis in rats.

### Effect of *A*. *orientale* on gut microecology in diabetic rats

**Fig 3:**
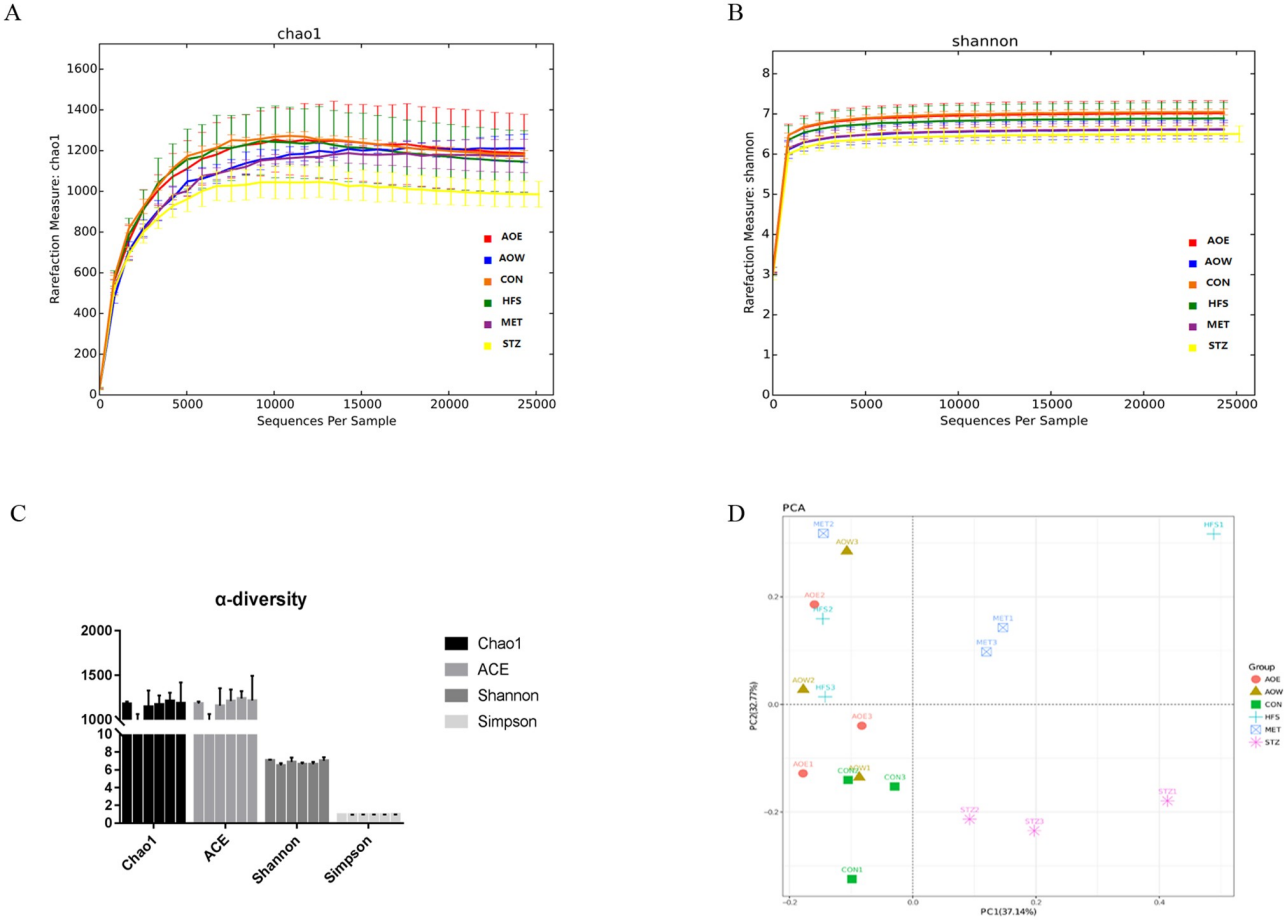
Effect of *A*. *orientale* on gut microecology. (A) Rarefaction curve of the Chao1 index. (B) Rarefaction curve of the Shannon index. (C) Alpha diversity analysis. (D) PCA analysis: each point represents a sample, drawn by the first principal component on the X-axis and the second principal component on the Y-axis, with one color per group. n = 3.

To investigate whether the treatment of *A*. *orientale* in diabetic rats is related to the changes in their gut microecology, 16S rRNA of 18 intestinal content samples were detected by high-throughput sequencing. A total of 968,160 effective reads were obtained from the samples of the six groups rats (*n* = 3). After obtaining the OTU abundance matrix, alpha diversity analysis was performed to calculate the diversity of each sample population. Rarefaction curves of the Chao1 and Shannon index indicated that the current sequencing depth of each sample was sufficient to reflect the microbial diversity contained in the community. The Chao1 index, ACE index, Shannon index and the Simpson index are commonly used metrics for alpha diversity analysis. The Chao1 index and ACE index focus on community richness, while the Shannon index and Simpson index take into account community uniformity. There was no obvious difference among the six groups (Fig 3).

**Fig 4:**
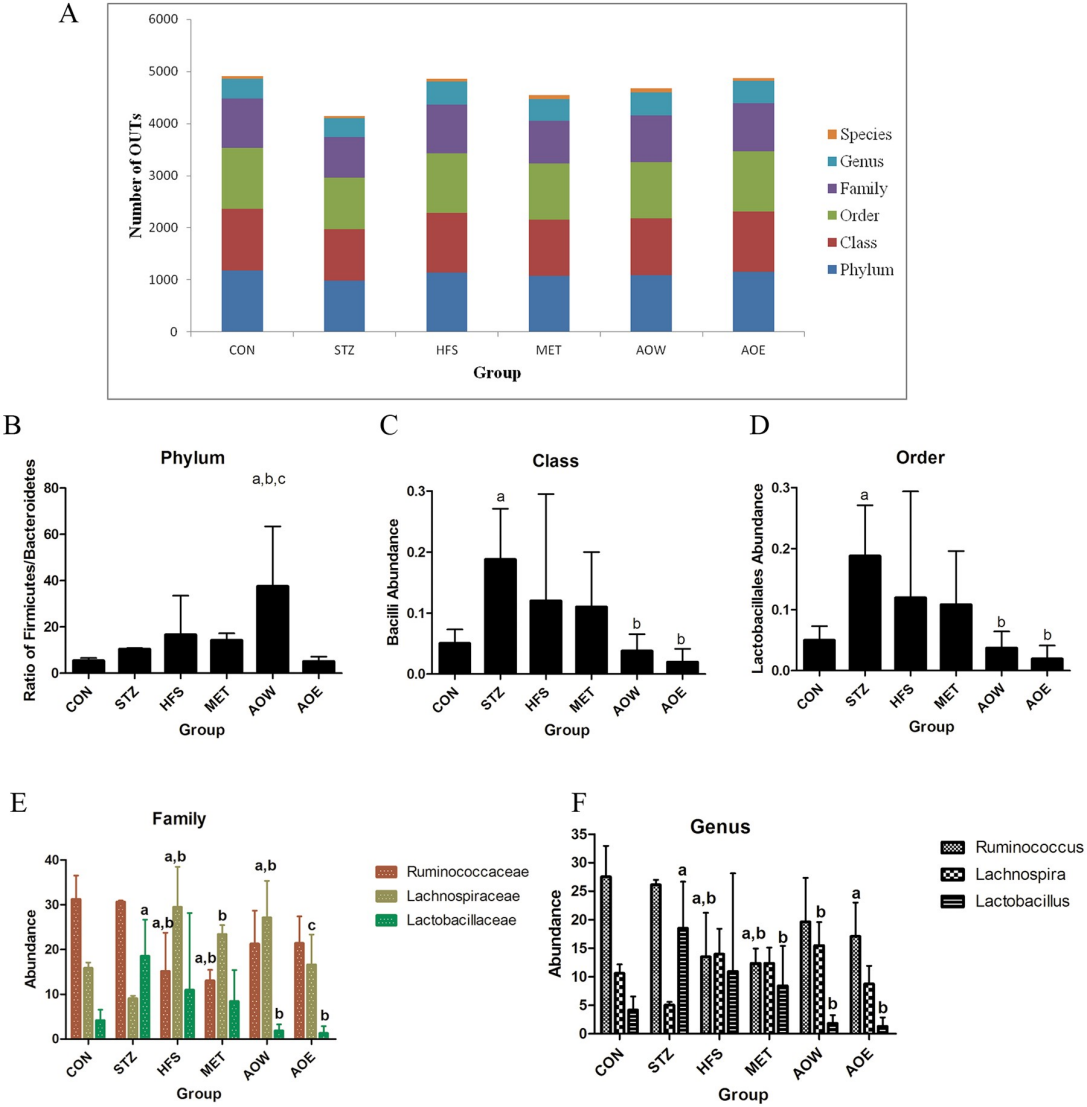
Taxonomic diversity of *A*. *orientale* on gut microecology in diabetic rats. (A) The number of OTUs and the classification status of different groups of samples. R-software was used to draw the histogram to visually compare the difference. (B) The ratio of *Firmicutes* to *Bacteroidetes* at the phylum level. (C) The relative abundance of *bacilli* in gut bacteria at the class level. (D) The relative abundance of *Lactobacillales* at the order level. (E) The relative abundance of *Ruminococcaceae*, *Lactobacillale* and *Lachnospiraceae* at the family level. (F) The relative abundance of *Ruminococcaceae*, *Lactobacillale* and *Lachnospiraceae* at the genus level. ^*a*^ *p* < 0.05 vs. the CON group, ^*b*^*p* < 0.05 vs. the STZ group, ^*c*^*p* < 0.05 vs. the HFS group. n = 3.

The OTU was divided and the classification status identification results were statistically analyzed. R-software was used to draw the histogram to visually compare the difference between the OTU number and the classification status of different groups of samples. The phylum, class, and order were the top three classifications with the largest OTU number, and the number of OTU classified to the species was the least (Fig 4A). The relative abundance of bacterial phylum types was compared among the six groups to further investigate the composition alterations in the gut microecology. *Firmicutes*, *Bacteroidetes*, *Proteobacteria*, *Verrucomicrobia* were the four most abundant bacterial phyla in the six groups. There was no obvious difference in the four phyla bacteria among the six groups, but the AOW group had the highest ratio of *Firmicutes* and *Bacteroidetes* compared with other groups (Fig 4B). At the class level, the STZ group displayed an increased relative abundance of *bacilli* in gut bacteria when compared with the CON group (Fig 4C). However, after treatment with *A*. *orientale*, the *bacilli* abundance was decreased notably. At the order level, gut bacteria from the STZ group displayed the same increasing trend of *Lactobacillales* abundance when compared with the other groups as the class level (Fig 4D). It’s a pity that there was no statistical significance between the CON_vs_HFS group and HFS_vs_AOE group in the above two levels.

At the family level, as shown in Fig 4E, the STZ group displayed an increased number of *Lactobacillaceae* in gut bacteria when compared with the CON group, and *A*. *orientale* administration had a notable decreasing effect on *Lactobacillaceae* abundance. The HFS and MET groups displayed a decreased relative abundance of *Ruminococcaceae* in the gut bacteria when compared with the CON group or STZ group. The finding most worth mentioning is the *Lachnospiraceae* relative abundance, which in the HFS, MET and AOW groups displayed an increased effect, while AOE treatment had a notable decreasing effect. In addition, there was no difference in *Lachnospiraceae* relative abundance among the AOE, CON and STZ groups. At the genus level, the STZ group displayed an increased relative abundance of *Lactobacillus* in the gut bacteria when compared with the CON group, but after treatment with *A*. *orientale* or MET, it was notably decreased (Fig 4F). The HFS group displayed a decreased relative abundance of *Ruminococcus* in the gut bacteria when compared with the CON group or STZ group, but there were no significant differences after treatment with *A*. *orientale* or MET (Fig 4F). Interestingly, there was no difference in the relative abundance of *Lachnospira* among the CON, STZ, HFS, MET and AOE groups.

**Fig 5:**
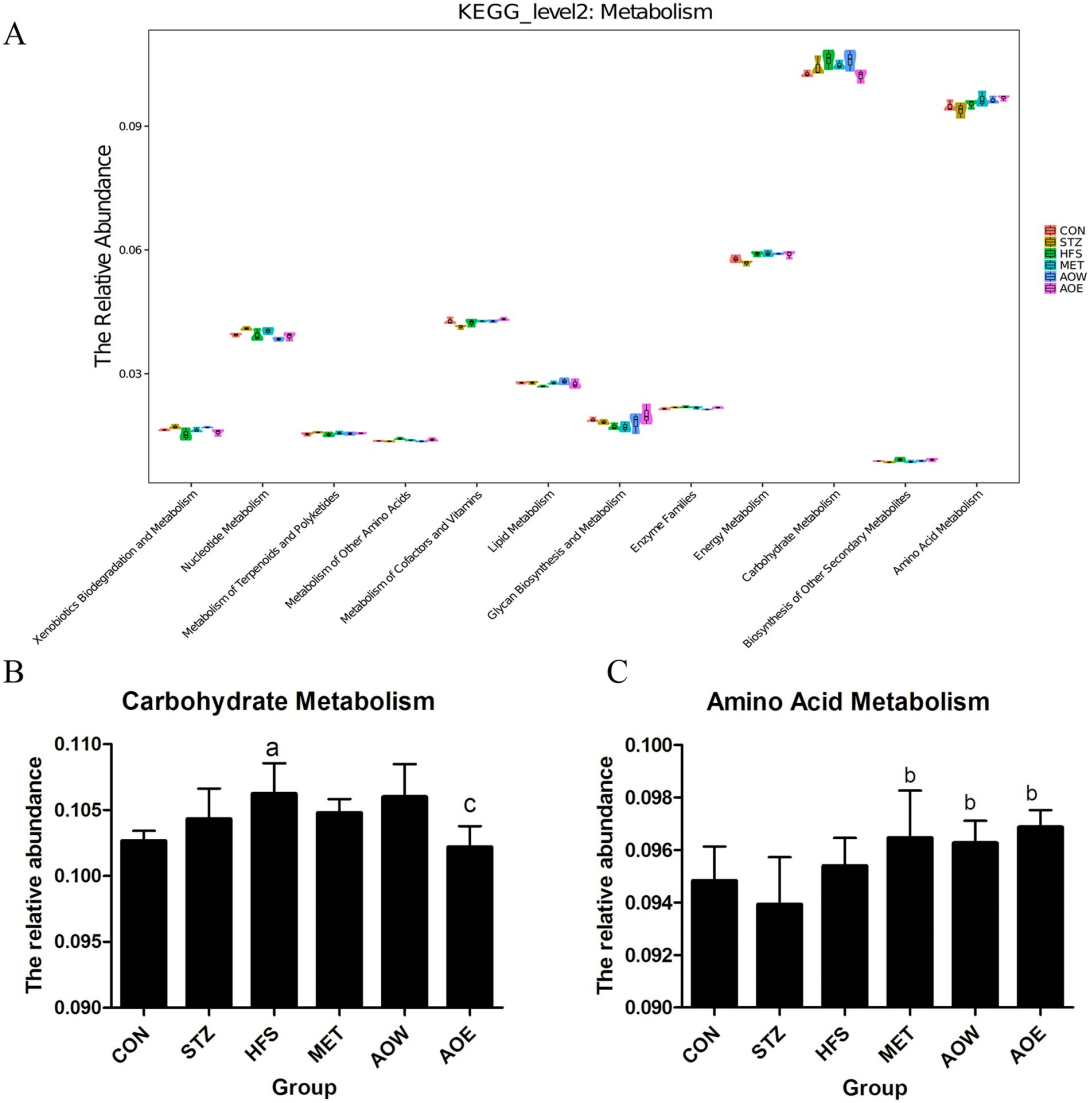
KEGG second level distribution predicted PICRUSt. Carbohydrate metabolism and amino acid metabolism were the two highest relative abundance metabolism pathways of the gut bacteria. (A) The KEGG level 2 PICRUSt. (B) Carbohydrate metabolism. (C) Amino acid metabolism. ^*a*^ *p* < 0.05 vs. CON group, ^*b*^*p* < 0.05 vs. STZ group, ^*c*^*p* < 0.05 vs. HFS group. n = 3.

In addition to the composition and structural analysis of the gut bacteria, the metabolic function of the bacteria is also a focus of gut microecology. In this study, the KEGG level 2 PICRUSt results showed that carbohydrate metabolism and amino acid metabolism were the two highest relative abundance metabolism pathways of the gut bacteria (Fig 5A). In the carbohydrate metabolism pathway, the relative abundance of gut bacteria in the HFS group increased when compared with the CON group, but after treatment with AOE, the relative abundance of gut bacteria significantly decreased (Fig 5B). In the amino acid metabolism pathway, the relative abundance of gut bacteria in the MET group, AOW group and AOE group had increased when compared with the STZ group (Fig 5C).

### Effect of *A*. *orientale* on the liver transcriptome in diabetic rats

To obtain the transcriptome dynamic profile of the hypolipidemic effect of AOE, four mRNA libraries were constructed of five liver samples from the CON, HFS, MET and AOE groups. RNA sequencing was performing on the Illumina HiSeq (TM) 2500 system. The Q30 values in all of the libraries were more than 94%. From the libraries of the CON, HFS, MET and AOE group rats, 214,205,422, 210,281,068, 230,221,008 and 218,810,100 high-quality reads were obtained respectively. The uniquely mapped rates in four of the libraries were all more than 91%. The results of the alignment of the gene regions showed that the ratio of reads mapped to exons were all over 90%, indicating that the prepared RNA libraries were of good quality and that the RNA sequencing data analysis results are reliable.

**Fig 6:**
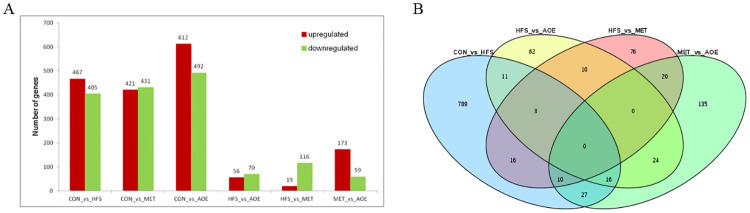
Statistical analysis of differentially expressed genes. (A) The number of significantly upregulated and downregulated genes in CON_vs_HFS, CON_vs_MET, CON_vs_AOE, HFS_vs_AOE, HFS_vs_MET and MET_vs_AOE. (B) Venn diagram of the counts of DEGs in CON_vs_HFS, HFS_vs_AOE, HFS_vs_MET and MET_vs_AOE (n = 5).

The analysis results showed that there were 3,321 DEGs in the six groups (CON_vs_HFS, CON_vs_MET, CON_vs_AOE, HFS_vs_AOE, HFS_vs_MET and MET_vs_AOE). The total number of DEGs of CON_vs_HFS and HFS_vs_AOE were 872 and 126, of which 467 and 56 were upregulated, whereas 405 and 70 were downregulated, respectively (Fig 6A). Venn diagrams were plotted to obtain the intersection and union among the four groups (CON_vs_HFS, HFS_vs_AOE, HFS_vs_MET and MET_vs_AOE) using the OmicShare tools, a free online platform for data analysis (www.omicshare.com/tools). The results showed that there were 789 specific DEGs in CON_vs_HFS, 62 specific genes in HFS_vs_AOE, 76 specific genes in HFS_vs_MET and 135 specific genes in MET_vs_AOE. There were 30 common DEGs between CON_vs_HFS and HFS_vs_AOE, 29 common DEGs between CON_vs_HFS and HFS_vs_MET, and 3 common DEGs among CON_vs_HFS, HFS_vs_AOE and HFS_vs_MET. There were no common DEGs among the four groups (Fig 6B).

### GO functional classification of DEGs

**Fig 7:**
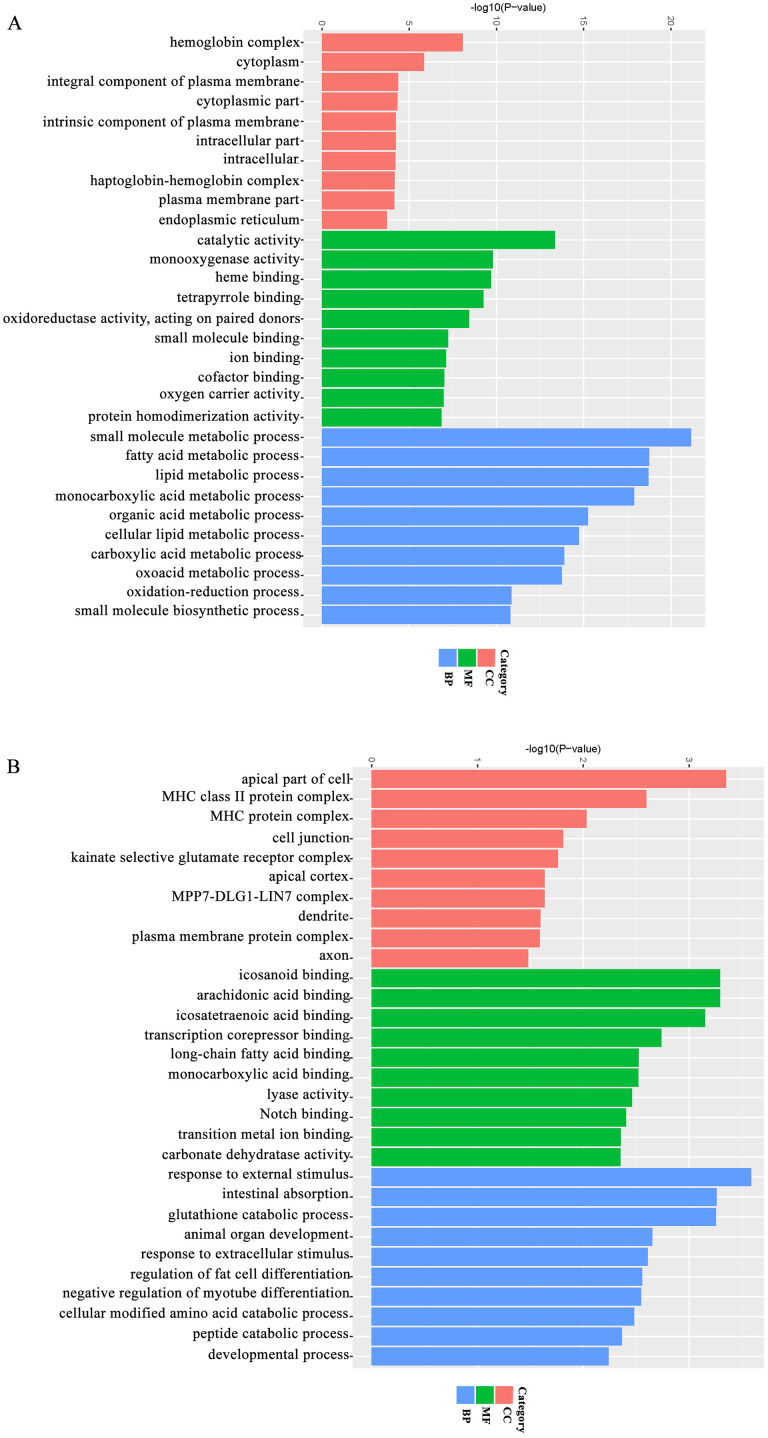
GO enrichment analysis histogram. (A) GO term of co-expressed DEGs in CON_vs_HFS group. (B) GO term of co-expressed DEGs in HFS_vs_AOE group.

GO classification can be divided into three functional categories: Biological Process (BP), Cellular Component (CC) and Molecular Function (MF). After obtaining the DEGs, the top 10 GO categories enrichment comparing the three groups (CON_vs_HFS and HFS_vs_AOE) were analyzed (Fig 7). In the BP categories, the most significant of the DEGs in the CON_vs_HFS group were assigned to small molecule metabolic process (142 genes, approximately 10% of total DEGs), fatty acid metabolic process (49 genes, approximately 19% of total DEGs), and lipid metabolic process single cell processes (106 genes, approximately 11% of DEG). In the CC categories, the most significant of the DEGs were classified into hemoglobin complex (8 genes, approximately 61% of the DEGs), cytoplasm (407 genes, approximately 5% of the DEGs), integral component of plasma membrane (48 genes, approximately 8% of the DEGs). In MF categories, the most significant of the DEGs were classified into catalytic activity (328 genes, approximately 6% of the DEGs),monooxygenase activity (24 genes, approximately 20% of the DEGs), heme binding (28 genes, approximately 18% of the DEGs); In the BP categories, the most significant of the DEGs in the HFS_vs_AOE group were assigned to response to external stimulus (25 genes, approximately 1% of total the DEGs), gut absorption (3 genes, approximately 12% of the total DEGs), glutathione catabolic process (2 genes, approximately 33% of the total DEGs); in the CC categories, the most significant of the DEGs were classified into apical part of cell (8 genes, approximately 3% of the DEGs), MHC class II protein complex (2 genes, approximately 15% of the DEGs), MHC protein complex (2 genes, approximately 8% of the DEGs); and in the MF categories, the most significant of the DEGs were classified into icosanoid binding (2 genes, approximately 33% of the DEGs), arachidonic acid binding (2 genes, approximately 33% of the DEGs), icosatetraenoic acid binding (2 genes, approximately 28% of the DEGs).

### KEGG classification of DEGs

**Fig 8:**
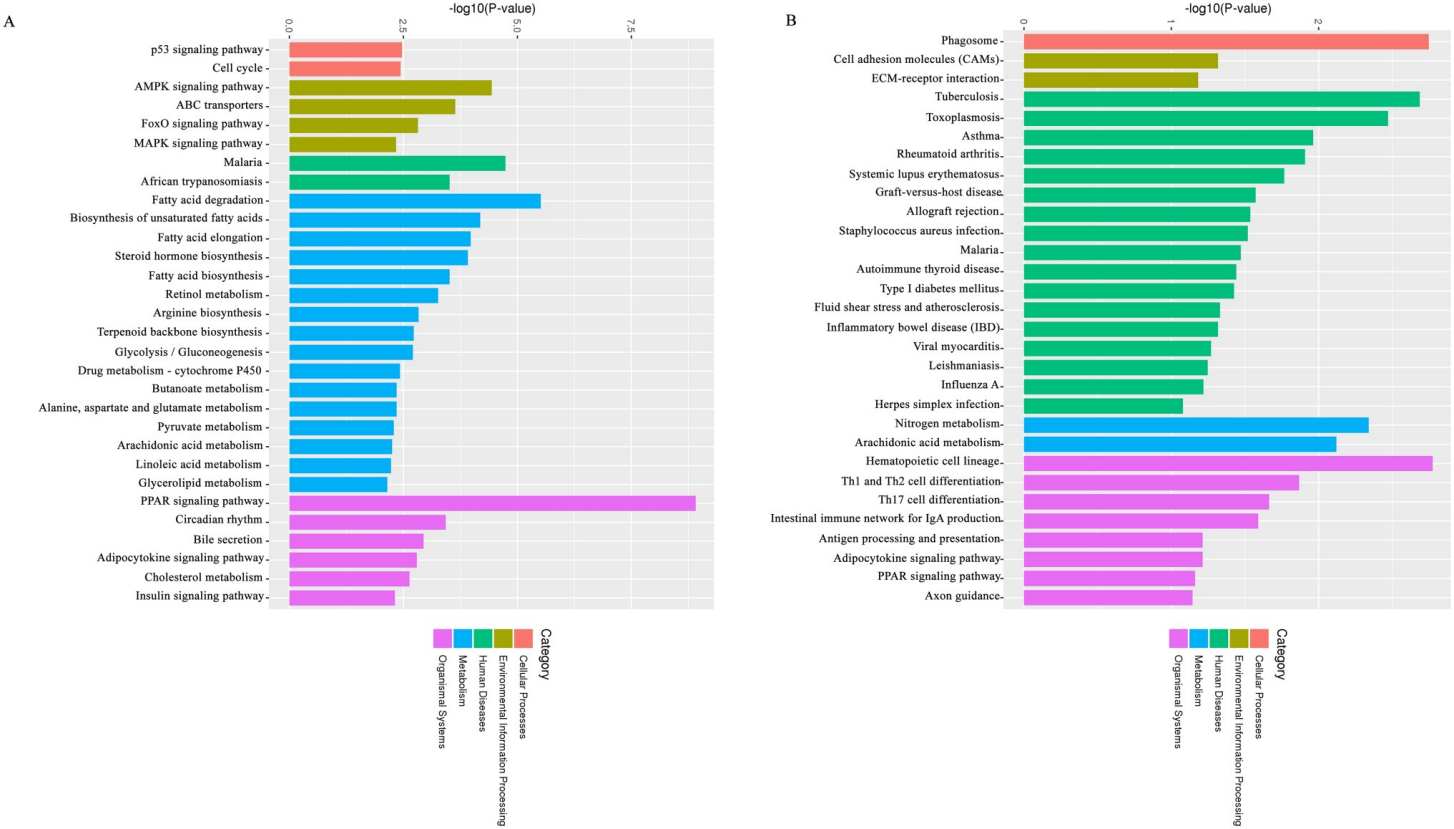
KEGG enrichment analysis histogram. (A) Pathway functional enrichment of co-expressed DEGs in CON_vs_HFS group. (B) Pathway functional enrichment of co-expressed DEGs in HFS_vs_AOE group.

To identify the pathogenesis of diabetes and the biological pathway activated during AOE treatment, the three groups of DEGs were mapped into the five categories (Cellular Processes, Environmental Information Processing, Human Diseases, Metabolism, Organismal Systems) of the KEGG database. The pathways with the lowest p-value and the top 20 pathways with the lowest False Discovery Rate (FDR) are shown in Fig 8. There were 60 known KEGG pathways in the CON_vs_HFS group. The top 10 pathways were the PPAR signaling pathway (19 DEGs), Fatty acid degradation (11 DEGs), Malaria (11 DEGs), AMPK signaling pathway (17 DEGs), Biosynthesis of unsaturated fatty acids (8 DEGs), and Fatty acid elongation (8 DEGs), Steroid hormone biosynthesis (11 DEGs), ABC transporters (9 DEGs), African trypanosomiasis (8 DEGs), and Fatty acid biosynthesis (5 DEGs). In addition, the statistically significant lipid metabolism pathways were Arachidonic acid metabolism, Linoleic acid metabolism, Glycerolipid metabolism, Glycerophospholipid metabolism, and Primary bile acid biosynthesis. DEGs in the HFS_vs_AOE group were only involved in 21 known KEGG pathways, of which many DEGs were involved in human diseases, such as Type I diabetes mellitus, Fluid shear stress and atherosclerosis, Asthma, Rheumatoid arthritis, Systemic lupus erythematosus, Graft-versus-host disease, Allograft rejection, Autoimmune thyroid disease, Inflammatory bowel disease (IBD), Tuberculosis, Staphylococcus aureus infection, Toxoplasmosis, and Malaria; followed by the Organismal Systems of Hematopoietic cell lineage, Th1 and Th2 cell differentiation, Th17 cell differentiation, Gut immune network for IgA production, and Nitrogen metabolism, Arachidonic acid metabolism at the metabolism level. For the HFS_vs_MET group, the DEGs were involved in a total of 29 known KEGG pathways, of which 14 pathways were classified into human diseases, such as Viral myocarditis, Type I diabetes mellitus, Primary immunodeficiency, Graft-versus-host disease, Allograft rejection, Autoimmune thyroid disease, Asthma, Rheumatoid arthritis, Systemic lupus erythematosus, Tuberculosis, *Staphylococcus aureus* infection, Toxoplasmosis, Epstein-Barr virus infection, and HTLV-I infection. This was followed by10 pathways of Organismal Systems in Vascular smooth muscle contraction, Pancreatic secretion, Insulin secretion, Ovarian steroidogenesis Gut immune network for IgA production, Antigen processing and presentation,Th1 and Th2 cell differentiation,Th17 cell differentiation, Hematopoietic cell lineage, and the IL-17 signaling pathway. The KEGG classification of DEGs showed that the pathways of AOE treatment were different from those of MET treatment.

**Fig 9:**
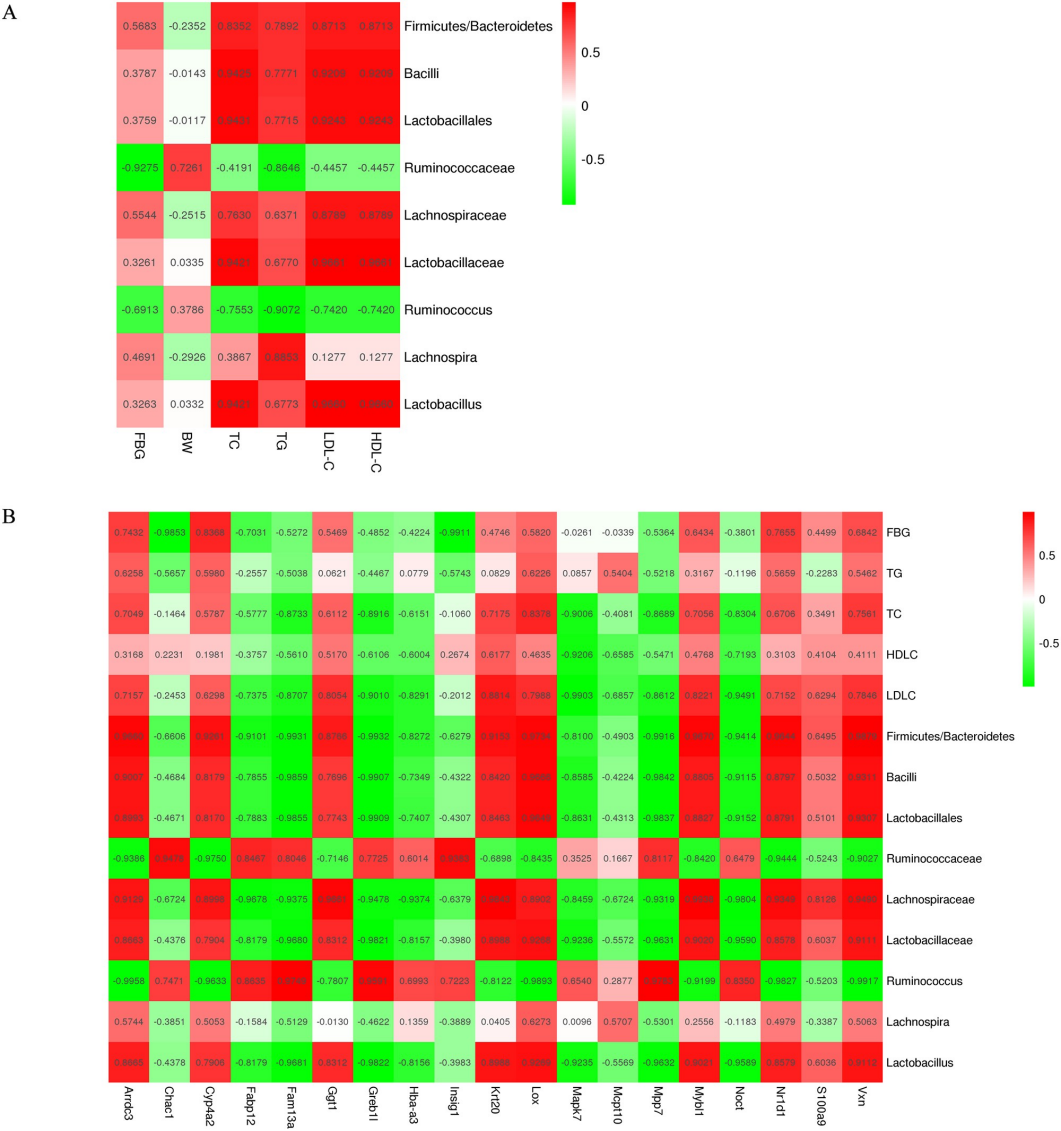
The Pearson correlation heatmap. (A) Correlation analysis between biochemical parameters and the different gut microbiota at the genus level. (B) Correlation analysis among biochemical parameters, the different gut microbiota and the 19 selected DEGs. A positive correlation is represented by red while a negative correlation is shown in green in the heat map.

To explore the interactive features between the DEGs of the liver transcriptome and the different gut microbiota (DGM) after the treatment with AOE, 19 DEGs between the CON_vs_HFS group and the HFS_vs_AOE group were selected to analyze the possible connection between the DGM and the DEGs (Table 1). The Pearson correlation analysis showed that several DEGs were significantly correlated with the DGM (Fig 9). Specifically, Arrdc3, Cyp4a2, Ggt1, Krt20, Lox, Mybl1, Nr1d1 and Vxn were positively correlated with *Firmicutes*/*Bacteroidetes*, Bacilli, *Lactobacillales*, *Lachnospiraceae*, *Lactobacillaceae* and *Lactobacillus* but negatively correlated with *Ruminococcaceae* and *Ruminococcus*; conversely, Fabp12, Fam13a, Greb1l, Mpp7 and Noct were negatively correlated with *Firmicutes*/*Bacteroidetes*, *Bacilli*, *Lactobacillales*, *Lachnospiraceae*, *Lactobacillaceae* and *Lactobacillus* but positively correlated with *Ruminococcaceae* and *Ruminococcus*; Insig1 and Chac1 were positively correlated with *Ruminococcaceae* and *Ruminococcus*; Mapk7 was negatively correlated with *Lactobacillaceae* and *Lactobacillus*; Hba-a3 was negatively correlated with *Lachnospiraceae*, and S100a9 was positively correlated with *Lachnospiraceae*; there was no obvious correlation between Mcpt10 and DGM, and there was no obvious correlation between *Lachnospira* and the 19 DEGs. Therefore, there is a significant correlation between the DEGs and DGM, and the treatment with AOE not only contributed to the DEGs of the liver transcriptome but also influenced the composition of the gut microbiota.

**Table 1:** The possible connection between the DEGs of the liver transcriptome and different gut microbiota after the treatment with AOE.

gene_id	Name	CON_vs_HFS _ log2fc	CON_vs_HFS_pval	HFS_vs_AOE_log2fc	HFS_vs_AOE_pval
ENSRNOG00000007947	Fam13a	-1.20127584	0.00182383	1.08665777	0.0003144
ENSRNOG00000002412	Mapk7	-Inf	0.03477149	Inf	0.0019114
ENSRNOG00000018760	Mpp7	-1.54415823	0.033892265	1.446105017	0.0184865
ENSRNOG00000014387	Chac1	-3.32515557	4.53425E-07	1.076356803	0.0436458
ENSRNOG00000006859	Insig1	-3.90168675	7.81859E-19	1.082190399	0.0132363
ENSRNOG00000049991	Mcpt10	-1.99047147	0.028280076	1.859241805	0.024636
ENSRNOG00000010799	Noct	-1.6026617	0.000285117	1.509174242	0.0017221
ENSRNOG00000023492	Greb1l	-1.42721832	2.61438E-05	1.361789787	0.0004628
ENSRNOG00000038825	Fabp12	-1.99693729	1.81747E-07	1.454191507	0.0072356
ENSRNOG00000045989	Hba-a3	-3.79286311	0.001462438	3.458842797	0.0002627
ENSRNOG00000014426	Lox	1.273966701	0.021787572	-1.68047127	0.0048201
ENSRNOG00000021669	Mybl1	2.128045034	0.014143272	-1.61791255	0.0406767
ENSRNOG00000045649	Arrdc3	1.596898139	1.12023E-07	-1.18187643	0.0001116
ENSRNOG00000030154	Cyp4a2	2.420294518	1.07669E-05	-1.06099037	0.0247949
ENSRNOG00000027139	Krt20	1.632926348	0.003394672	-1.64311171	0.0009927
ENSRNOG00000021663	Vxn	1.649218031	0.016859805	-1.44041241	0.0253653
ENSRNOG00000047697	Ggt1	1.967731427	0.00867844	-1.4849093	0.0222422
ENSRNOG00000009329	Nr1d1	1.737400351	3.25719E-08	-1.16116203	0.0002606
ENSRNOG00000011483	S100a9	1.931674927	0.001516338	-1.10779264	0.0323183

### Transcriptional level verification of Insig1

**Fig 10:**
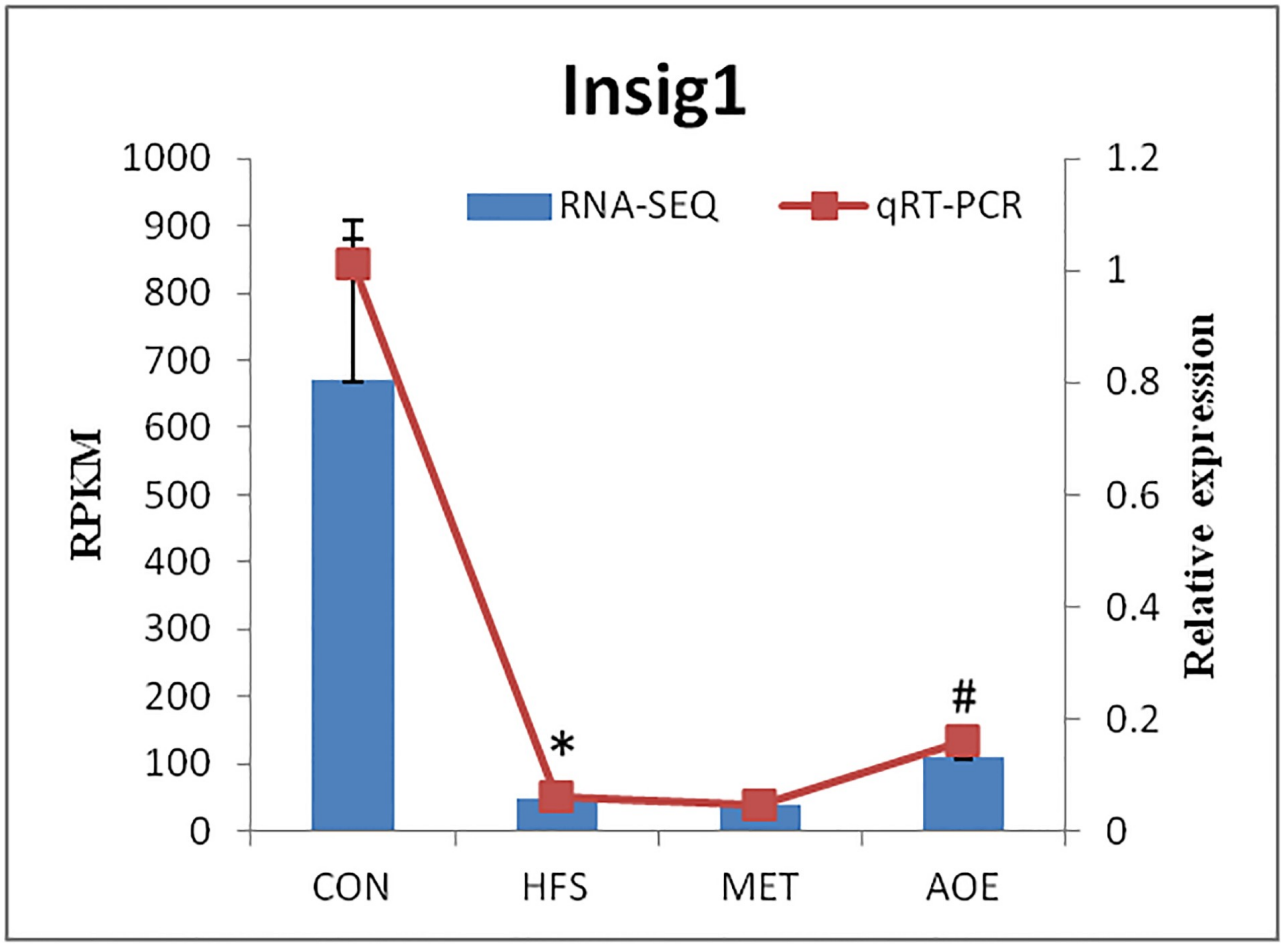
The expression levels of the Insig1 were verified by qRT-PCR (n = 3). * *p* < 0.05 vs. CON group, ^*#*^ *p* < 0.05 vs. HFS group.

Recent studies have indicated that Insig1 plays an important role in the regulation of fatty acid and cholesterol metabolism [37]. To verify the accuracy of the Illumina sequencing, we selected Insig1 to verify the differences obtained from transcriptome sequencing by qRT-PCR. The results indicate that the gene expression level of Insig1 verified by qRT-PCR revealed similar trend when compared with the RNA-Seq samples (Fig 10).

### Characterization of major active compounds in AOE

**Fig 11:**
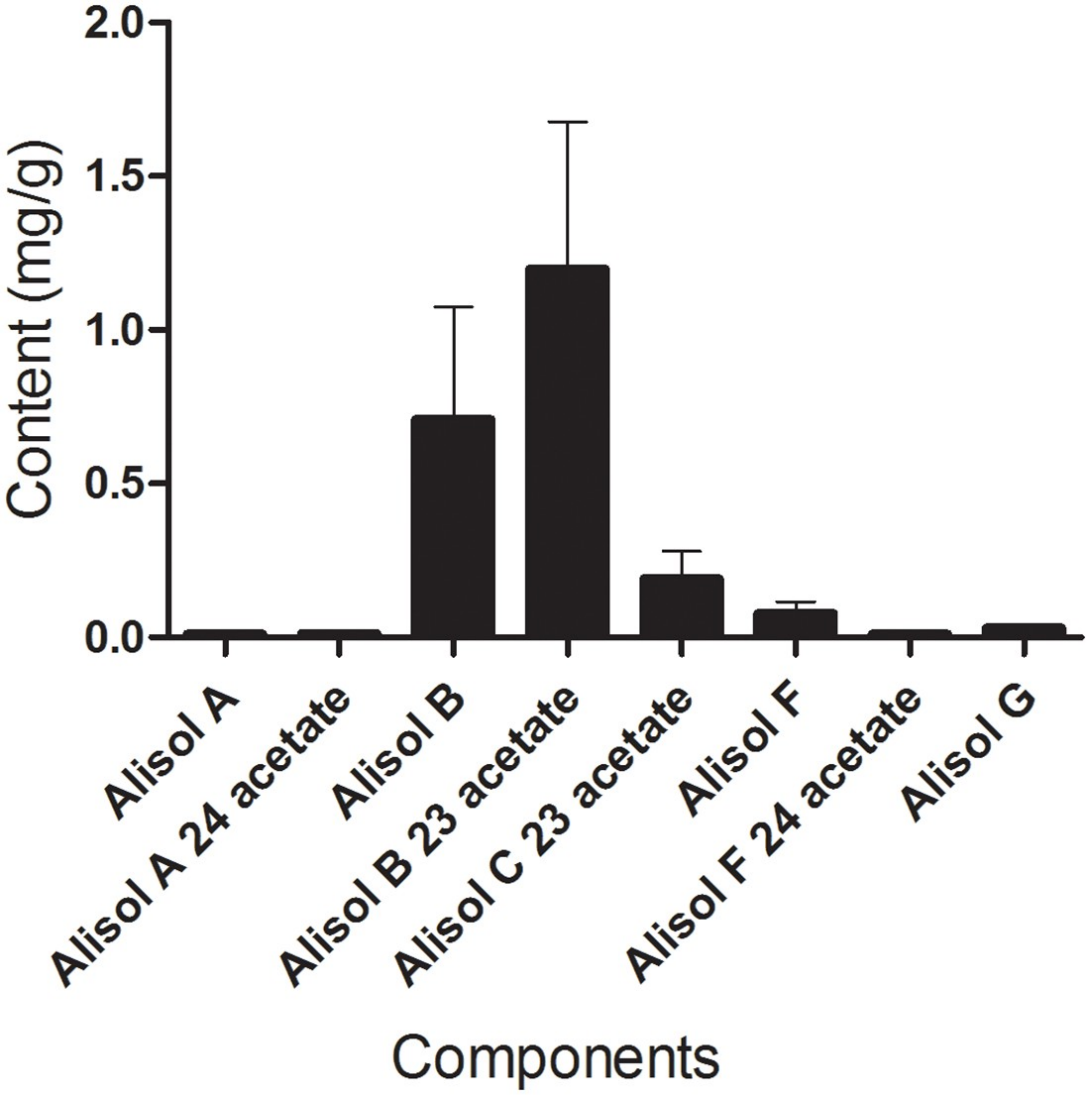
The characterization of major active compounds in AOE.

The contents of the eight compounds of alisol A, alisol A 24-acetate, alisol B, alisol B 23-acetate, alisol C 23-acetate, alisol F, alisol F 24-acetate, and alisol G determinate by UPLC-QQQ-MS are shown in Fig 11. The results indicated that all eight compounds could be detected in the AOE. Alisol B 23-acetate, alisol B and alisol C 23-acetate were the major compounds, which may be the active compounds of the hyperlipidemic effect of AOE.

## Discussion

In this study, the HFS group rats were given a high fat feed and exhibited persistent hyperglycemia during the experiment compared with the CON and STZ group rats. After two weeks of treatment, the serum LDL-C level decreased significantly in the AOE group compared with the HFS group. The macroscopic and pathological analysis of the livers showed that the liver morphology was ameliorated after administration of AOE. These results indicate that AOE could promote the recovery of fatty liver in HFS rats. Previous studies have reported significant reduction in serum TC and LDL-C atherogenic indexes in high-fat diet model mice or rats following administration of the triterpene fraction of *A*. *orientale* [38, 39]. Therefore, our results are basically consistent with the reduction in serum LDL-C level as previously reported with other high fat model rodents.

The key roles of gut microbiota in the etiology and development of various physiological diseases, and changes in the microbiota are related to a numbers of human diseases, such as allergic diseases, autoimmune encephalitis, atherosclerosis, colorectal cancer, obesity, and diabetes [40, 41]. *Ruminococcaceae* and *Lachnospiraceae* are dominant families in energy metabolism, and they are strongly correlated with adipokine levels. There is a positive correlation between the *Ruminococcaceae* and insulin, C-peptide, and leptin levels [42]. *Lactobacillus* is a kind of probiotic in the human gastrointestinal tract for preventing or treating T2DM [43].

In this study, there were significant differences in the abundance of *Lactobacillaceae*/*Lactobacillus*, *Ruminococcaceae*/*Ruminococcus* and *Lachnospiraceae*/*Lachnospira* among the six groups. *A*. *orientale* was found to normalize the high-fat diet and streptozotocin-induced gut microbiota dysbiosis. Particularly, *A*. *orientale* ameliorated gut microecology changes in the abundance of the three taxa back to normal status (e.g., AOE increased *Ruminococcus*, AOW enhanced *Lachnospira*, but both of them decreased *Lactobacillus*, which was significantly increased in the STZ group). In this study, FBG and serum TG level were significantly negative correlated with the abundance of *Ruminococcaceae*/*Ruminococcus*, and the serum TC level exhibited pronounced positive correlations with the abundance of *Lactobacillaceae*/*Lactobacillus*. Based on these findings, we believed that the hypolipidemic effect of *A*. *orientale* in diabetic rats is associated with an alteration of gut microbiota composition.

The pathogenesis of obesity is a complex process involving the regulation of many genes. To identify the process of obesity and the DEGs after AOE treatment, the total RNAs of the liver from CON rats, HFS rats, MET and AOE-treated group rats were extracted and RNA-sequenced. According to the DEGs analysis in the liver transcriptome of the four groups of rats, there were 19 significant DEGs among the groups. The results showed that ten coexpressed DEGs were significantly upregulated and nine coexpressed DEGs were significantly downregulated in AOE_vs_HFS and HFS_vs_CON. Among the 19 DEGs, there were five genes (Insig1, CYP4A2, Fabp12, nocturnin and Nr1d1) involved in the pathways of lipid metabolism. Insulin-induced gene 1 protein (Insig1) mediates feedback control of cholesterol synthesis by controlling SCAP and HMGCR [44–47]. Cytochrome P450 4A2 (CYP4A2) catalyzes the omega- and (omega-1)-hydroxylation of various fatty acids [48, 49]. Fatty acid-binding protein 12 (Fabp12) may play a role in lipid transport [50, 51]. Nocturnin is a circadian deadenylase, which plays an important role in posttranscriptional regulation of metabolic genes under circadian control and exerts a rhythmic posttranscriptional control of genes necessary for metabolic functions [52–56]. Moreover, Nuclear receptor subfamily 1 group D member 1 (Nr1d1) is a transcriptional repressor, which coordinates the circadian rhythm and metabolic pathways in a heme-dependent manner [57]. It regulates genes involved in metabolic functions, including lipid and bile acid metabolism [58, 59], adipogenesis [60], glucose homeostasis [61] and the macrophage inflammatory response [62, 63]. These differentially expressed genes provide new target proteins for the molecular mechanism of the hypolipidemic effect of *A*. *orientale*.

In current study, the FBG level were significantly negative correlations with the expression level of insig1, and the body weight exhibited strongly positive correlations with the expression level of insig1, The serum TC, HDL-C and LDH-C level were significantly negative correlations with the expression level of nocturnin, and the serum TC, TG, HDL-C, LDH-C level, and the body weight were positively correlated with the expression level of Nr1d1. Based on these findings, we believe that changes in the expression levels of these DEGs may be the molecular basis for the reduction of blood lipid levels in diabetic rats after AOE treatment.

Cholesterol is an extremely important biological molecule that has roles in membrane structure as well as being a precursor molecule for the synthesis of the steroid hormones, the bile acids, and vitamin D [64]. In the pathway of cholesterol biosynthesis, the rate limiting enzyme is 3-hydroxy-3-methylglutaryl-CoA (HMG-CoA) reducatase (HMGCR) [65]. In the pathway of cholesterol metabolism, CYP7A1 is the rate limiting enzyme in the primary pathway of bile acid synthesis pathway [66, 67]. Our results revealed that oral administration of AOE did not affect the mRNA levels of HMGCR and CYP7A1, but increase the mRNA and protein expression levels of insig1. Insig1 is one of the insig encoding genes, the other one is insig2. Insig1 gene expression is highest in human liver while Insig2 gene expression is ubiquitous. Both insig proteins can cause ER retention of the SREBP/SCAP complex, which results in decrease of the intracellular cholesterol content. Expression of Insig1 has also been shown to be regulated by several members of the nuclear receptor family including PPARδ, PXR and CAR. In addition to their role in regulating sterol-dependent gene regulation, both Insig proteins activate sterol-dependent proteasomal degradation of HMGCR. In present study, there were no changes in the expression levels of insig2, Scap, Srebf1, Srebf2 and Hmgcr between the HFS group and AOE group. Based on these findings, it suggested that the hypolipidemic effect of A. *orientale* in diabetic rats was attributed to increasing levels of Insig1 transcription.

There were certain limitations to this study. First, two weeks of treatments with metformin or *A*. *orientale* might not be long enough to change the liver function, nor to make significant changes in gut microecology. Further study should be carried out to investigate what would happen to these indicators if the administration time was prolonged. In addition, further research was needed to determine which active compound in AOE plays the leading role in hypolipidemic effect, and the corresponding hypolipidemic pathways about Insig1.

## Conclusion

Based on the results of RNA-Seq transcriptomics, we identified some DEGs involved in the pathogenesis of diabetes and elucidated the potential molecular mechanism, by which AOE exerted its protective effects in diabetic rats. The active ingredients in AOE exerted hypolipidemic effect potentially by acting on Insig1. Finally, we presented a hypothetical model for how AOE played its role of hypolipidemic effects that will help us to understand more about the molecular ecological mechanisms.

## Supporting information

S1 Data (XLSX)

S1 Fig (TIF)
