# Supplementary figures and images for: Hypolipidemic effect of Alisma orientale (Sam.) Juzep on gut microecology and liver transcriptome in diabetic rats
Source: PLoS One. 2020 Oct 9;15(10):e0240616. doi: 10.1371/journal.pone.0240616 (PMC7546448; doi:10.1371/journal.pone.0240616)

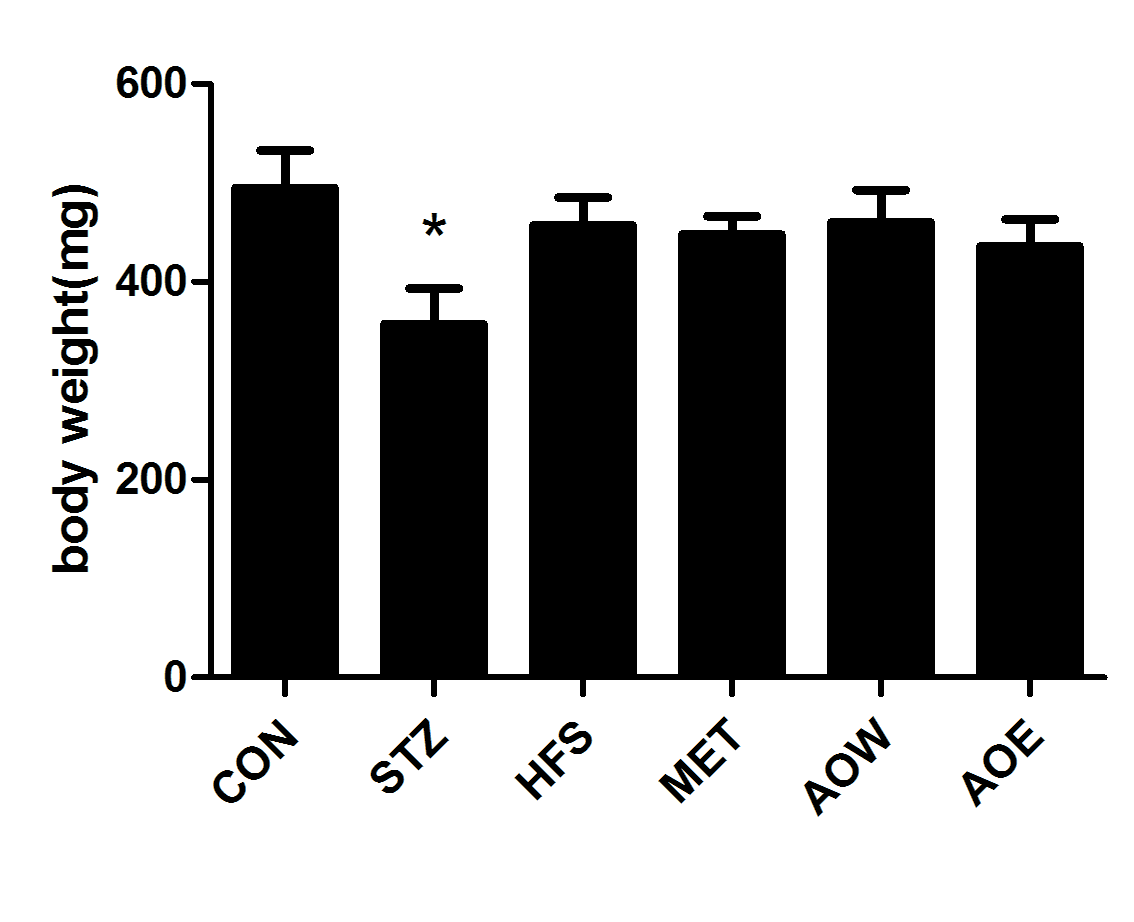

Supplement: S1 Fig — (TIF) [file pone.0240616.s002.tif]
